# Fertility in classical galactosaemia, a study of *N*-glycan, hormonal and inflammatory gene interactions

**DOI:** 10.1186/s13023-018-0906-3

**Published:** 2018-09-19

**Authors:** Hugh-Owen Colhoun, Estela M. Rubio Gozalbo, Annet M. Bosch, Ina Knerr, Charlotte Dawson, Jennifer Brady, Marie Galligan, Karolina Stepien, Roisin O’Flaherty, C. Catherine Moss, P. Peter Barker, Maria Fitzgibbon, Peter P. Doran, Eileen P. Treacy

**Affiliations:** 1 Department of Paediatrics, Trinity College, Dublin, Ireland; 2 Department of Pediatrics and Department of Clinical Genetics, Maastricht University Medical Centre, P. Debyelaan 25, PO Box 5800, 6202 AZ Maastricht, The Netherlands; 3 Department of Pediatrics, Division of Metabolic Disorders, Academic Medical Center, University of Amsterdam, Amsterdam, The Netherlands; 4 National Centre for Inherited Metabolic Disorders, Temple Street Children’s University Hospital and Mater Misericordiae University Hospital, Dublin, Ireland; 5 Department of Endocrinology, University Hospitals Birmingham NHS Foundation Trust, B15 2TH, Birmingham, UK; 6 Department of Clinical Biochemistry and Diagnostic Endocrinology, The Mater Misericordiae University Hospital, Dublin, Ireland; 7 University College Dublin, Belfield, Dublin 4, Ireland; 8 Mark Holland Metabolic Unit, Salford Royal NHS Foundation Trust, M6 8HD, Manchester, UK; 9 NIBRT GlycoScience Group, National Institute for Bioprocessing, Research and Training, Mount Merrion, Blackrock, Co, Dublin, Ireland; 10 Core Genomics Facility, Conway Institute, University College, Dublin, Ireland; 11 Core Biochemical Assay Laboratory (CBAL), Clinical Biochemistry, Box 232, Cambridge University Hospitals NHS Foundation Trust, Cambridge Biomedical Campus, Hills Road, Cambridge, CB2 0QQ UK; 12 National Centre for Inherited Metabolic Diseases, The Mater Misericordiae University Hospital, Eccles St, Dublin, 7 Ireland

**Keywords:** Classical galactosaemia, Infertility, Glycan modifier genes

## Abstract

**Background:**

Classical Galactosaemia (CG) (OMIM #230400) is a rare inborn error of galactose metabolism caused by deficiency of the enzyme galactose-1-phosphate uridylyltransferase (GALT). Long-term complications persist in treated patients despite dietary galactose restriction with significant variations in outcomes suggesting epigenetic glycosylation influences. Primary Ovarian Insufficiency (POI) is a very significant complication affecting females with follicular depletion noted in early life. We studied specific glycan synthesis, leptin system and inflammatory gene expression in white blood cells as potential biomarkers of infertility in 54 adults with CG adults (27 females and 27 males) (age range 17–51 yr) on a galactose-restricted diet in a multi-site Irish and Dutch study. Gene expression profiles were tested for correlation with a serum Ultra-high Performance Liquid Chromatography (UPLC)-Immunoglobulin (IgG)-*N*-glycan galactose incorporation assay and endocrine measurements.

**Results:**

Twenty five CG females (93%) had clinical and biochemical evidence of POI. As expected, the CG female patients, influenced by hormone replacement therapy, and the healthy controls of both genders showed a positive correlation between log leptin and BMI but this correlation was not apparent in CG males. The strongest correlations between serum leptin levels, hormones, G-ratio (galactose incorporation assay) and gene expression data were observed between leptin, its gene and G-Ratios data (r_s_ = − 0.68) and (r_s_ = − 0.94) respectively with lower circulating leptin in CG patients with reduced IgG galactosylation. In CG patients (males and females analysed as one group), the key glycan synthesis modifier genes *MGAT3* and *FUT8,* which influence glycan chain bisecting and fucosylation and subsequent cell signalling and adhesion, were found to be significantly upregulated (*p* < 0.01 and *p* < 0.05) and also the glycan synthesis gene *ALG9* (*p* < 0.01). Both leptin signalling genes *LEP* and *LEPR* were found to be upregulated (p < 0.01) as was the inflammatory genes *ANXA1* and *ICAM1* and the apoptosis gene *SEPT4* (*p* < 0.01).

**Conclusions:**

These results validate our previous findings and provide novel experimental evidence for dysregulation of genes *LEP*, *LEPR*, *ANXA1*, *ICAM1* and *SEPT4* for CG patients and combined with our findings of abnormalities of IgG glycosylation, hormonal and leptin analyses elaborate on the systemic glycosylation and cell signalling abnormalities evident in CG which likely influence the pathophysiology of POI.

## Background

Classical galactosaemia (CG) (OMIM #230400) is a rare autosomal recessive inborn error of galactose metabolism caused by deficiency of the enzyme galactose-1-phosphate uridylyltransferase (GALT; EC 2.7.7.12). This condition occurs with a prevalence ranging from 1:16,000 to 1:60,000 in Europe and USA. Life-long galactose restricted diet is the only treatment currently available for this condition. Although this treatment is life-saving in the neonate, long-term complications including cognitive impairment, neurological and speech abnormalities, and fertility problems in female patients, persist in treated adult patients despite early diagnosis and initiation of treatment [1–8]. Ovarian damage and subfertility with primary ovarian insufficiency (POI) is a major complication for females causing a very significant disease burden. The exact timing of the ovarian insult and its pathophysiology remains poorly understood [3, 6].

A key limitation to improving care and personalised therapy for affected individuals for this rare disease is the lack of insight into the pathophysiology and the lack of reliable and accurate biomarkers that can predict the risk of developing disease complications and can monitor the outcome of therapeutic interventions.

Moreover, outcomes can differ even in siblings with the same *GALT* genotype, illustrating the complex nature of this condition with the presence of recognised significant epigenetic effects on the fundamental glycosylation pathways involved in galactosaemia [9–11].

The toxic build-up of the GALT substrate galactose-1-phosphate (Gal-1-P) and its metabolites are proposed to be central to the pathophysiology of the ongoing complications [6]. In the neonatal acute intoxicated phase, very high ambient levels of Gal-1-P can inhibit a number of metabolic processes. Elevated Gal-1-P is a known competitive substrate for inositol monophosphatase 1 (IMPase1) and various glycosyltransferases and also inhibits UDP-hexose pyrophosphorylases [6, 12]. In addition, over-restriction of galactose in the long-term may contribute to the disease phenotype by further depleting UDP-galactose in susceptible individuals, potentially disrupting glycosylation dependent pathways [13, 14]. Down-regulation of the key P13K/Akt signalling pathway has also been recently reported in the GALT deficient mouse model [15].

It has long been recognised that the measurement of red blood cell (RBC) Gal-1-P in neonatal blood samples from untreated patients prompts the initial treatment of the Gal-1-P intoxicated neonate and is an important diagnostic marker. However, monitoring RBC Gal-1-P and urinary galactitol concentrations have not generally been considered to be reliable prognostic indices of long-term outcomes [4, 13, 16, 17].

Previous investigations by our group have explored the mechanisms by which abnormal galactosylation of glycoproteins may contribute to the ongoing pathophysiology in galactosaemia and its complications. Of relevance to fertility, recent studies have not demonstrated any differences in Follicle stimulating hormone (FSH) glycosylation pattern or bioactivity in females with CG [18, 19]. A high percentage of females with CG have Anti-mullerian hormone (AMH) levels below the detection limit refecting a low ovarian reserve, however the glycosylation status of AMH has not been studied to date in CG [20]. IgG is the predominant circulating glycoprotein in serum and is very well studied [21]. We developed a glyco-analysis of Immunoglobulin G (IgG) and studied the incorporation of galactose into IgG in galactosaemia patients in comparison to healthy controls using an automated hydrophilic interaction ultra-high performance liquid chromatography (HILIC-UPLC) *N*-glycan analytical method for serum IgG, to monitor *N*-glycan processing defects in galactosaemia [22, 23].

We previously evaluated the impact of several genes on abnormal glycosylation in galactosaemia patients and noted significant altered expression of a number of relevant *N*-glycan biosynthesis genes in peripheral blood mononuclear cells (PBMCs) from adult galactosaemia patients involving four key *N*-glycan biosynthesis genes*: ALG9*, *MGAT3, FUT8* and *B4GALT1* which correlated with the IgG variant profiles also identified in the study [9]. Lending further weight to this finding, a large European population genome wide association study using liquid chromatography mass spectrometry (LC-MS) to measure IgG glycoprotein variant characteristics has recently demonstrated that polymorphisms of the glycan genes encoding the glycosyltransferases (*ST6GAL1, B4GALT1, FUT8* and *MGAT3*) represent the most important loci associated with variation in IgG traits [24].

In addition to altered glycosylation, we previously identified a number of key central signalling pathways affected in T lymphocyte cell studies to include the unfolded protein response (UPR) pathway, the inositol signalling pathway, oxidative phosphorylation, and inflammatory pathways [25, 26]. We also observed dysregulation of pathways in leptin metabolism, a key hormone in the Hypothalamic-Pituitary-Gonadal (HPG) axis which stimulates release of luteinising hormone (LH), follicle stimulating hormone (FSH) and oestrogen [27, 28].

The majority of adult females with CG have POI which is a spectrum varying from absent or delayed pubertal development, primary amenorrhoea in adolescents, secondary amenorrhoea to irregular or premature menopause [29]. POI in females with CG is identifiable by elevated follicle stimulating hormone (FSH) and LH levels and decreased oestradiol levels [29]. FSH levels have been found to be significantly elevated from four months of life [6, 29, 30]. The precise timing of the severe decrease in primordial ovarian follicles and the absence of intermediate and Graafian follicles, which suggest a maturation arrest, is not clear [6, 29, 30]. A recent paper has suggested that follicles are maintained in early childhood, but commence depletion as early as the 1st year of life [31]. Different mechanisms have been proposed to explain these changes to include prenatal toxicity of galactose and its metabolites, including apoptosis, abnormal signalling pathways and abnormal gonadotropin function [29, 30, 32].

There is limited data available for assessing fertility in CG adult males, though studies have reported delayed onset of puberty [6]. In a study of 26 CG males, the prevalence of cryptorchidism was noted to be higher in CG males than in the general population and the CG patients had subtle decreases in testosterone, inhibin B and sperm concentration [33].

As it is not practical to study temporal ovarian or testicular gene expression in patients, in this study we have aimed to investigate the expression of key glycan and other relevant genes in GC patients in a cohort of patients from a three-site study, (Irish and two Dutch national galactosaemia cohorts) using accessible PBMCs (peripheral blood mononucleocytes) and correlated these findings with the patient endocrine markers and *N*-glycome glycosylation status as measured by the IgG *N-*glycome.

## Results

Clinical Characterisation: Table 1 illustrates the study subject characteristics: age, gender and genotypes.

**Table 1:** Patient Characteristics

Group	Controls	Galactosaemia	
Ethnicity	Irish	Irish	Dutch
Patients	*n* = 16	*n* = 36	*n* = 18
Age (years)	20–40	17–51	18–47
Gender	9F, 7 M	15F, 21 M	12F, 6 M
Genotype (nucleotide annotation)	NA	31: (c.563A > G/c.563A > G) 2: c.568A > G/c.580 T > C 1: c.563A > G/c.997C > T 1: c.563A > G/c.379.A > G) 1: c.563A > G/unknown	10: (c.563A > G/c.563A > G) 3: (c.584 T > C/c.687G > T) 1: (c.563A > G/c.584 T > C) 1: (c.563A > G/c.400delT) 1: (c.563A > G/del exon 1_10) 1: (c.956A > C/c.956A > C) 1: Unknown
Genotype (protein variant)	NA	31: p.Q188R/p.Q188R 2: Q188R/p.F194 L 1: p.Q188R/p.R333W 1: p.Q188R/p.K127E, 1: p.Q188R/unknown	10: (p.Q188R/p.Q188R) 3: (p.L195P/p.K229 N) 1: (p.Q188R/p.L195P) 1: (p.Q188R/p.T134 fs) 1: (p.Q188R/del exon 1_10) 1: (c.956A > C/c.956A > C) 1: Unknown

### Endocrine studies

Patient FSH, LH, oestradiol, testosterone serum concentrations and reference intervals are illustrated in Table 2. Of the 27 CG females, 25 had clinical and biochemically documented POI (92.59%, age range 18–42 yr). 22 of these patients were on hormone replacement therapy (HRT).

**Table 2:** Patient Hormone Results

	LH (IU/L)	FSH (IU/L)	Oestradiol (pmol/L)	LH (IU/L)	FSH (IU/L)	Testosterone (nmol/L)(Males only)
Normal reference ranges^a^	F: 1.8–11.8	F: 3.0–8.1	F: 92–921	1.4–6.5	0–10.9	7.1–31.1
	M: 7.6–89.1	M: 2.6–16.7	M: 139–2382			
	L: 0.6–14.0	L: 1.4–5.5	L: 92–1145			
	CG Females (*n* = 24)			CG Males (*n* = 18)		
Median	11.57	32.85	460 (*n* = 9) < 92 (*n* = 14) No data (*n* = 1)	4.00	2.00	18.80
Mean	14.94	40.89	500.67 (*n* = 9) < 92 (*n* = 14) No data (*n* = 1)	5.51	8.40	19.98
Range	0.5–40.50	0.5–120	< 92–967	0.5–9.4	0.5–9.3	10–31.6
SD	38.43	12.52	N/A	6.10	2.521.47	5.49

^a^Reference ranges are provided for healthy females and males for LH, FSH and oestradiol for comparison to CG patients, with the females divided into three menstrual stages: Follicular Phase (F), Mid Cycle Phase (M) and Luteal Phase (L). The normal reference ranges were provided from the Investigator site accredited diagnostic laboratory. For CG patients, median, mean and range data are provided with standard deviation (SD)-no menstrual stage data was available. Oestradiol levels less than 92 pmol/L were noted as undetectable according to the limits of the assay. Hormone data was available for 38 of the 54 patients

It was not possible to determine which phase of the menstrual cycle patients were in when the hormone samples were taken. Inappropriately raised FSH (> 20 IU) in the context of suppressed oestradiol in females below the age of 40 is an indicator of POI. Oestradiol results were available for 23 CG females, 14 of whom had low or undetectable levels (< 92 pmol/mL). All but one of these 14 females were on HRT. However synthetic oestradiol levels are not detected by the assay used. There were no significant hormone abnormalities noted in male CG patients (Table 2).

Table 3 illustrates the leptin data for available CG subjects (*n* = 37) and healthy controls (*n* = 20). Circulating serum leptin data from both male and female galactosaemia cohorts were determined. The mean serum leptin level was lower in both CG males and females in comparison to healthy controls although this only reached statistical significance in males (*p* < 0.03). There was no significant difference noted in the soluble leptin receptor (sObR) levels between CG patients and healthy controls in either gender group. Leptin levels corrected for BMI were correlated using linear regression analysis with the IgG galactose incorporation (G0/G1 and G0/G2) ratios and with LH, FSH levels for both genders, and with testosterone for males.

**Table 3:** Serum Leptin levels in CG vs healthy controls and correlations with hormone, G-ratios and *ANXAI* gene expression

Serum Leptin^a^					
Gender	*Group*	*n*=	Mean ± SD	Range	*p*-value
Male	CG	18	2.6 ± 1.86	0.10–6.60	0.031
	Healthy Control	10	7.40 ± 5.60	0.70–18.80	
Female	CG	19	13.22 ± 9.47	1.18–33.12	0.099
	Healthy Control	10	20.10 ± 12.53	3.60–45.44	
Spearman Correlations - Serum Leptin Corrected for BMI^b^					
Variable 1	Variable 2	Gender	*n*=	*p*-value	Correlation Coefficient
Leptin, BMI corrected	G0/G1	Male and Female	26	0.0001	− 0.681
Leptin, BMI corrected	G0/G2	Male and Female	26	0.002	− 0.588
Leptin, BMI corrected	Testosterone	Male	11	0.026	0.664
Leptin, BMI corrected	FSH	Male and Female	25	0.027	0.441
Leptin, BMI corrected	ANXA1	Female	17	0.012	0.593

^a^Leptin data was available for 37 of 54 patients. The *p*-values of this section show results of the difference in means of serum leptin levels in CG vs healthy control (Mann Whitney U test)

^b^Correlations between serum leptin corrected for BMI vs other variables in combined and separate gender cohorts

The Spearman correlations between abnormal *N-*glycosylation as measured by G ratios and leptin are shown in Table 3. For those CG subjects that had RBC Gal-1-P levels performed (*n* = 22), we identified a direct positive correlation between Gal-1-P and G-ratios in the combined group (r_s_ = 0.699, *p* < 0.0005 [G0/G1], r_s_ = 0.666, *p* < 0.005 [G0/G2] (data not shown).

We observed a strong negative correlation between leptin and G-ratios in the combined CG group, indicating lower circulating leptin in patients with reduced IgG galactosylation (Table 3). We also observed a significant correlation between BMI corrected leptin levels and testosterone levels in CG males with a less significant correlation evident with FSH levels in the CG combined group (males and females analysed together).

**Fig. 1:**
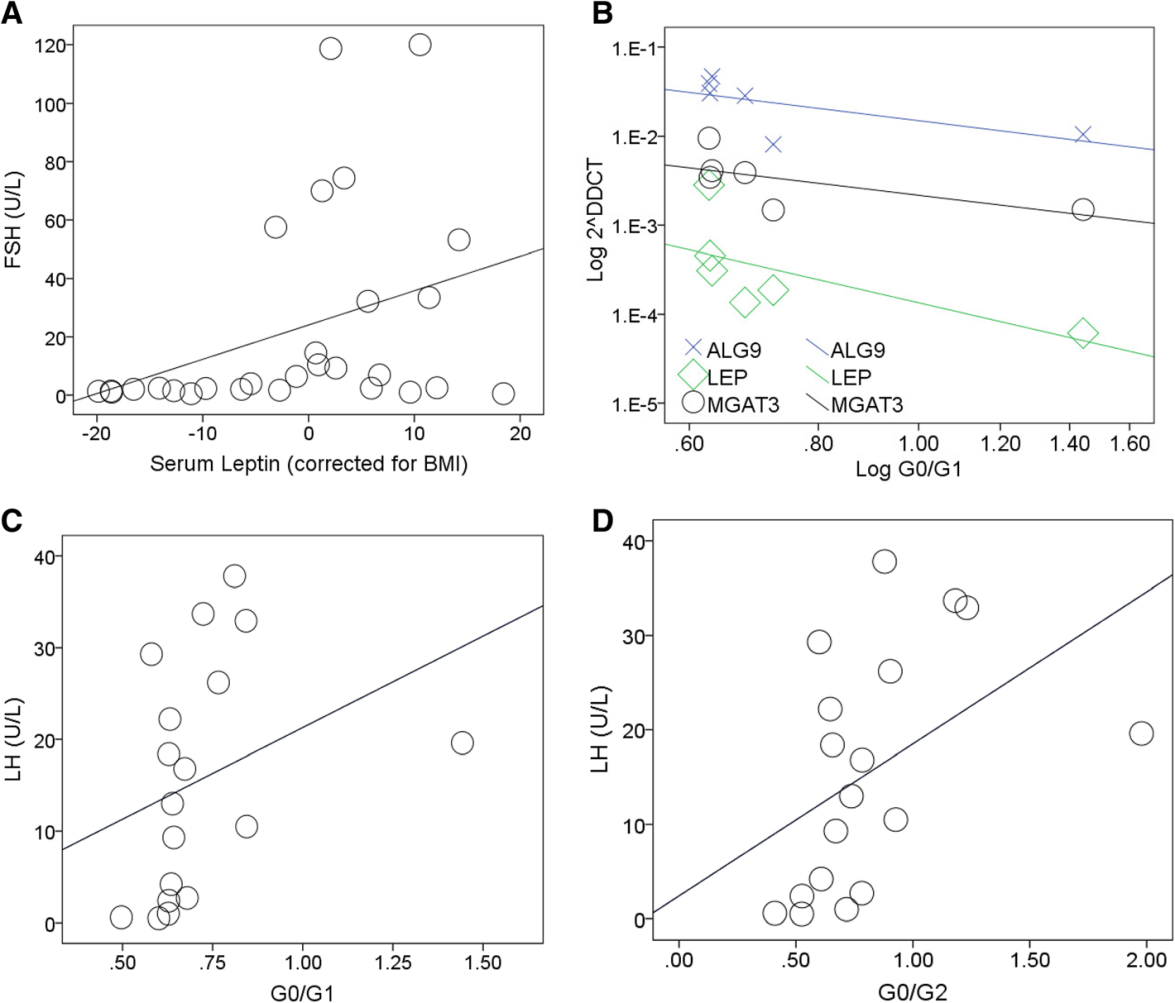
Hormones, G Ratio and gene correlations in galactosaemia. The strength of the association is denoted by r_s_ (Spearman’s ρ), 1 being a perfect positive correlation, − 1 being a perfect negative correlation. The *p*-value (2-tailed) for the association is indicated. The sloping line is the best fit line and indicates the direction of the correlation. **a** FSH levels correlate positively with BMI corrected leptin in the CG combined group (*n* = 28: r_s_ = 0.445, *p* < 0.05). **b** ALG9, LEP and MGAT3 expression correlate negatively with G-ratios (G0/G1) in CG females (*ALG9*: *n* = 18, r_s_ = − 0.600, *p* < 0.01. *MGAT3*: *n* = 13, r_s_ = − 0.687, *p* < 0.05. *LEP*: *n* = 6, r_s_ = − 0.943, *p* < 0.01). **c** LH levels correlate positively with G-ratios (G0/G1) in CG females (*n* = 18, r_s_ = 0.529, *p* < 0.05). **d** LH levels correlate positively with G-ratios (G0/G2) in CG females (*n* = 18, r_s_ = 0.608, *p* < 0.01)

As shown in Fig. 1, LH levels correlated with G0/G1 and G0/G2 ratios (r_s_ = 0.529, *p* < 0.05 and r_s_ = 0.608, *p* < 0.01) respectively in CG females (Fig. 1). There was no significant correlation evident between FSH levels and G-ratios in either genders (data not shown).

Figure 1 illustrates a positive correlation between FSH and leptin in the combined group (leptin corrected for BMI: r_s_ = 0.441, *p* < 0.05).

Leptin is known to correlate positively with BMI in both healthy males and females [32].

**Fig. 2:**
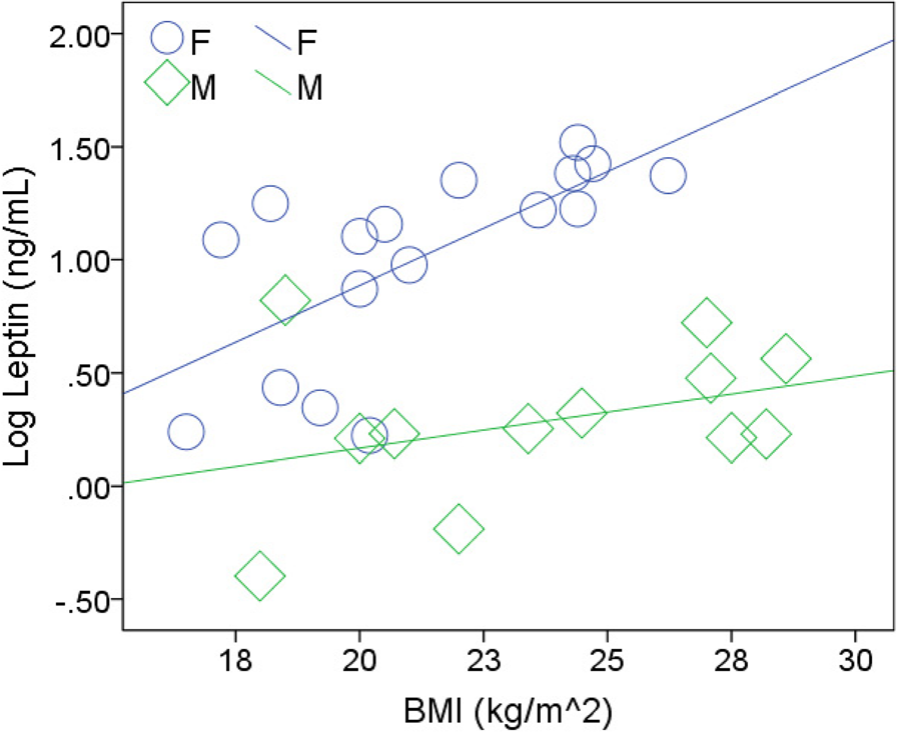
Correlation of log leptin and BMI in both CG males and females. Associations are measured with r_s_ and *p* values as described previously. Serum leptin correlates positively with BMI in healthy males and females [32]. This correlation was apparent in the CG female cohort (*n* = 17, r_s_ = 0.730, *p* < 0.005) but not in CG males (*n* = 12, r_s_ = 0.301, NS)

As shown in Fig. 2, the association between log leptin and BMI evident in CG females was absent in CG males (Fig. 2).

### Gene expression studies

**Fig. 3:**
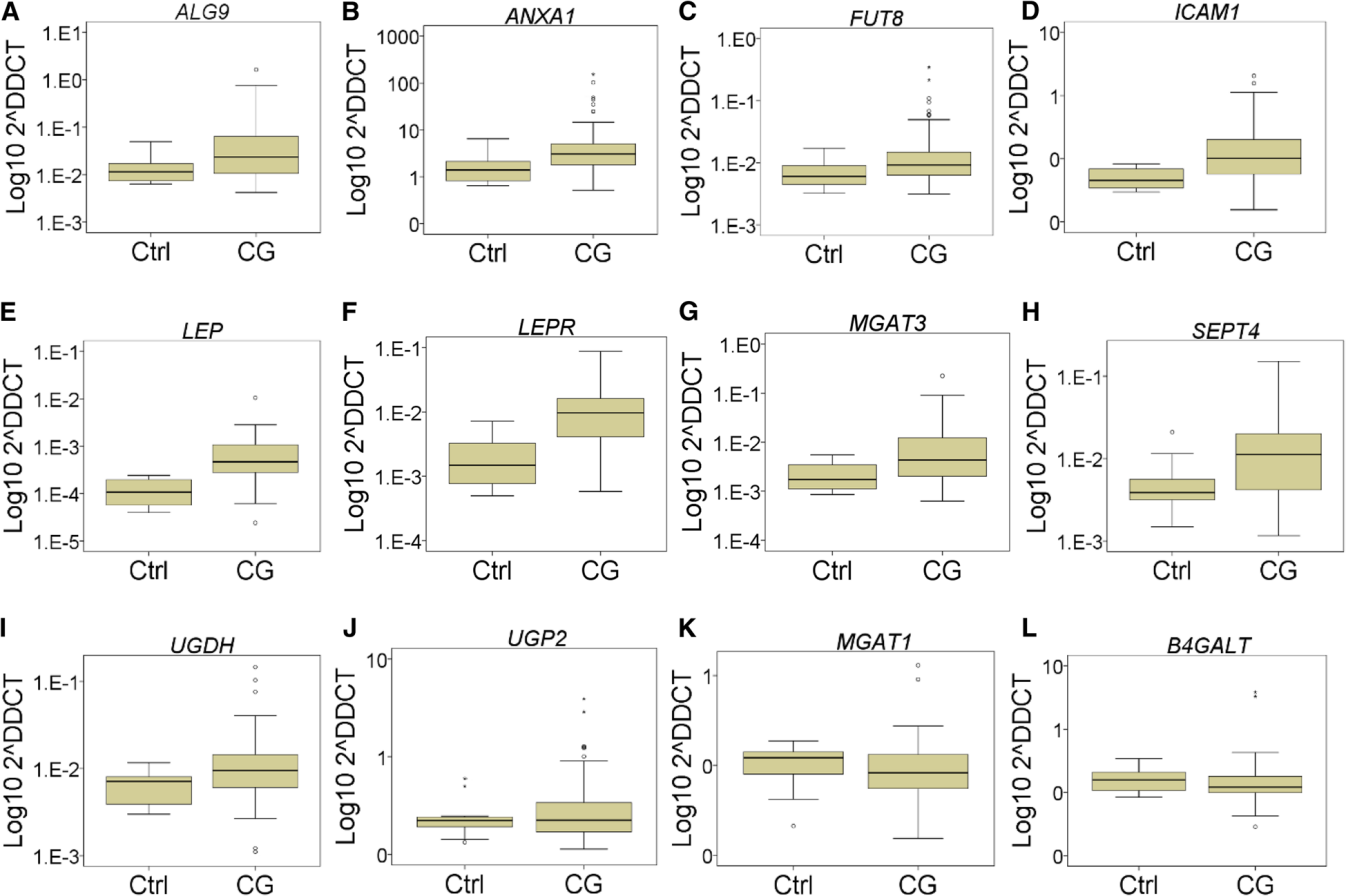
Boxplots of PBMC gene expression in CG combined group vs healthy controls (Ctrl). Each boxplot is titled with the relevant gene. The y-axis represents the 2^-ΔCT^ value of gene expression (Applied Biosystems). The y-axis scale has been log transformed to the base 10 for clarity. Fold change (RQ) and 2^ΔΔCT^ calculated with DataAssist (Applied Biosystems). Differences in expression between CG and Ctrl groups as calculated by The Mann Whitney U test, giving a p value which has been Benjamini-Hochberg False Discovery Rate (FDR) adjusted. Boxes indicate median (middle), 25th (bottom) and 75th (top) percentiles. Error bars indicate 1.5 times the interquartile range. Outliers are indicated with small circles or asterisks (extreme outliers). **a** *ALG9* upregulated in CG vs Ctrl (*n* = 52 vs 16, RQ = 5.97, *p* < 0.005). **b** *ANXA1* upregulated in CG vs Ctrl (*n* = 54 vs 16, RQ = 6.21, *p* < 0.01). (**c**) *FUT8* upregulated in CG vs Ctrl (*n* = 50 vs 16, RQ = 4.13, *p* < 0.05). **d** *ICAM1* upregulated in CG vs Ctrl (*n* = 53 vs 16, RQ = 4.79, *p* < 0.005). **e** *LEP* upregulated in CG vs Ctrl (*n* = 22 vs 10, RQ = 8.88, *p* < 0.005). **f** *LEPR* upregulated in CG vs Ctrl (*n* = 47 vs 16, RQ = 6.38, *p* < 0.0005). **g** *MGAT3* upregulated in CG vs Ctrl (*n* = 47 vs 16, RQ = 6.10, *p* < 0.01). **h** *SEPT4* upregulated in CG vs Ctrl (*n* = 52 vs 16, RQ = 3.62, *p* < 0.01). **i** *UGDH* upregulated in CG vs Ctrl (*n* = 46 vs 16, RQ = 2.66, *p* < 0.05). **j** UGP2 in CG vs Ctrl (*n* = 53 vs 16, not significant [NS]). **k** MGAT1 (*n* = 53 vs 16, NS). **l** B4GALT (*n* = 54 vs 16, NS)

In this current study we observed significant upregulation of the glycan synthesis genes, and inflammatory and leptin signaling genes; *ALG9*, *ANXA1, FUT8, ICAM1, LEP, LEPR, MGAT3, SEPT4* and *UGDH* (Fig. 3) in PBMC cells of the CG combined group (both genders). The expression of the genes *B4GALT1, MGAT1, UGP2* was not significantly altered in this group.

The key dysregulated glycan synthesis genes (*ALG9, MGAT3* and *FUT8*) correlate positively with each other (*p* < 0.05^− 4^) in CG which could be expected as they share the same glycan synthesis pathway.

*MGAT3* was noted to correlate directly with the expression of the inflammatory/apoptosis genes *ANXA1* and *SEPT4* (*p* < 0.05^− 4^) in CG, (combined group). The two inflammatory markers (ANXA1 and ICAM1*)* are positively correlated (*p* < 0.05^− 4^) in CG. Of note, increased expression of *FUT8* (indicating dysregulation of the core fucosylation pathway) was significantly correlated with *LEPR* expression (*p* < 0.0005) in CG.

There was no gender dependent difference in expression of any of the genes studied as determined by multivariate analysis of 2^-ΔCT^ values. The same finding was apparent between males and females of the healthy control group.

## Discussion

In this study, we sought firstly to validate identified glycan gene expression markers identified from our earlier studies in this larger multi-site study. We then aimed to introduce further novel inflammatory and putative fertility-linked expression markers and to correlate these expression profiles with the concurrent hormonal profiles of the study subjects with a view to further study the pathophysiology and fertility issues observed in CG.

*ALG9* (the gene that encodes α-1,2-mannosyltransferase*)* was shown to be overexpressed in this larger male and female CG cohort (*n* = 54 Irish and Dutch subjects: 5.97 fold, *p* < 0.01) in agreement with our earlier study [9]. α-1,2-mannosyltransferase catalyses the transfer of mannose from Dol-P-Man to lipid-linked oligosaccharides in *N-*Glycan assembly. This enzyme is involved in the addition of the seventh and ninth mannose to the growing *N*-glycan chain. This enzyme may be overexpressed in galactosaemia as cellular stresses increase the rate of glycan assembly in the endoplasmic reticulum (ER) which leads to improved levels of mono- and di-galactose glycan species (G1 and G2) downstream of the processing chain in the Golgi. In our previous cell studies, we observed that the activity of this enzyme is very responsive to galactose intoxication [26].

*MGAT3* (the gene that encodes β-1,4-mannosyl-glycoprotein 4-β-*N*-acetylglucosaminyltransferase) was the most significantly dysregulated glycan synthesis gene in this study, upregulated 6.1 fold in CG males & females vs controls (*p* < 0.01). The importance of this gene in *N-*glycosylation is demonstrated in the recent Genome-Wide Association Study (GWAS) [24] whereby variation in *MGAT3* was observed to be significantly associated with IgG glycan variant phenotypes. *MGAT3* is responsible for synthesis of complex hybrid type glycans in the endoplasmic reticulum. In the GWAS study, ratios of structures with bisecting GlcNAcs to structures without bisecting GlcNAcs were associated with SNPs at the *MGAT3* locus. Abnormal expression of this gene in our earlier study was associated with decreased bisecting GlcNAcs (decreased core fucosylated, non-fucosylated and monogalactosylated glycans) in CG [9]. It is considered that bisecting GlcNAcs influence glycan processing and glycan adhesion [34].

We observed that *FUT8* (that encodes alpha-1,6–fucosyltransferase) was upregulated in the CG combined group vs healthy controls (4.12 fold, *p* < 0.05)) in this study. This was more significant than in our previous smaller study and correlates with the findings of increased glycan core fucosylation, which we have observed previously [9]. The findings also indicate this gene as a significant modifier of glycan phenotype variation (ratios of fucosylated over non-fucosylated glycan structures) as noted in the GWAS study [24].

**Fig. 4:**
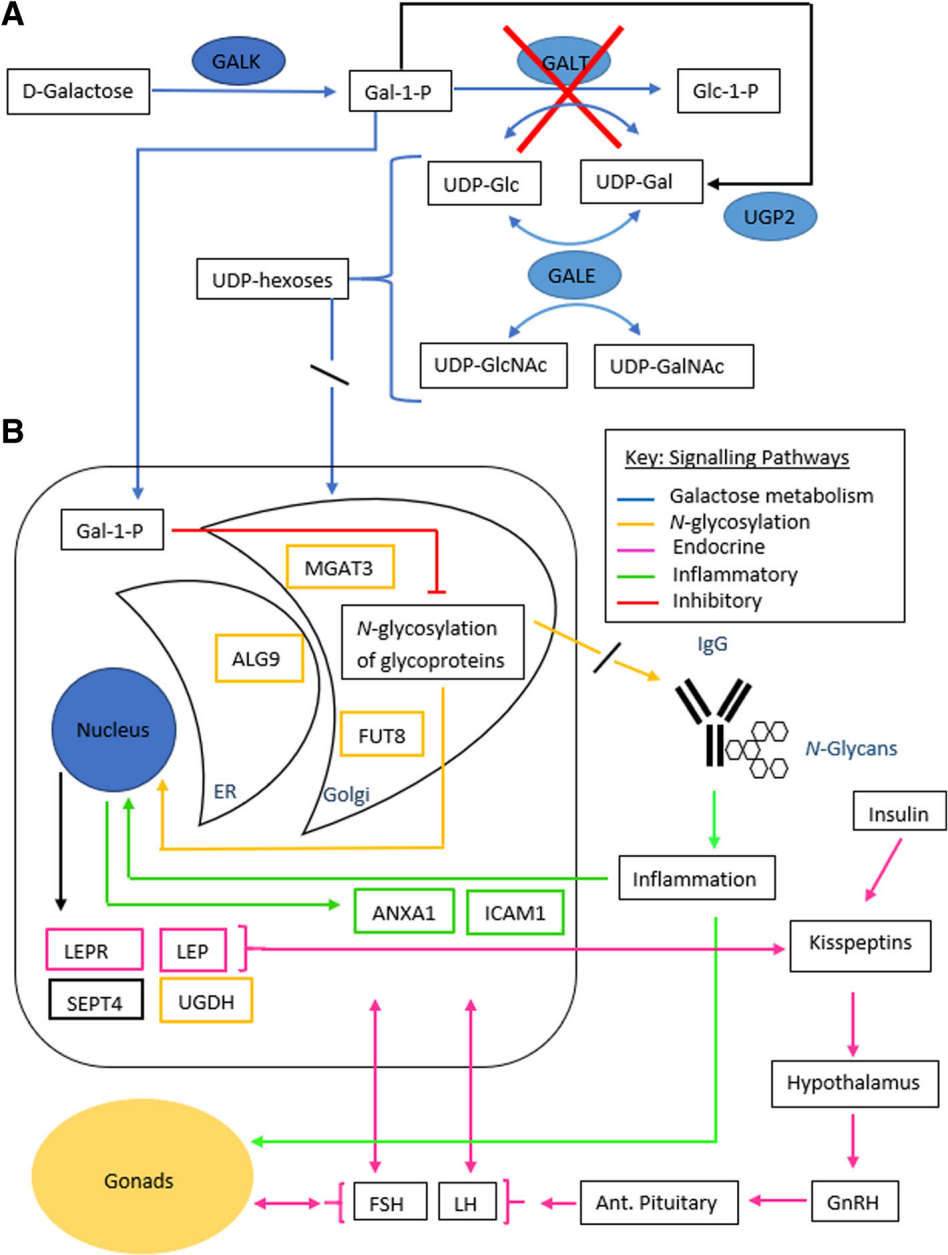
Galactose metabolism, glycosylation and inflammatory cascades in galactosaemia. **a** In galactosaemia, the Leloir pathway is disrupted with the absence of GALT, leading to an increase of Gal-1-P and disruption of UDP-hexose turnover. **b** Increased Gal-1-P levels may lead to cellular toxicity and ER stress, with competitive inhibition of glycosyltransferases and inhibition of UDP-hexose pyrophosphorylases. T [6, 11–13]. Disrupted glycosylation may lead to an upregulation of glycan synthesis genes. Abnormal glycosylation of IgG may lead to an activated immune conformation [36] and increased expression of anti-inflammatory genes. Leptin expression, influenced by abnormal glycosylation, cross signals with the HPG axis and gonadal function [52, 53]

The group of Jumbo-Lucioni et al. have demonstrated in a *Drosophila* galactosaemia model that the *UGDH* (UDP-Glucose 6-dehydrogenase) gene is upregulated and considered to be a modifier/rescue glycosylation gene in *drosophila* [35]. We included this gene in our analysis and we also observed upregulation 2.6 fold in the CG combined group (*p* < 0.05). This change may be a response to reduced substrate from disruption of UDP-Glc turnover via the GALT enzyme in the Leloir pathway (Fig. 4). Another potential salvage gene that we included is *UGP2* (UDP-glucose pyrophosphorylase 2). We did not note any significant difference in expression of this gene between CG patients and healthy controls.

Of the glycan synthesis genes, *ALG9* overexpression correlated with the overexpression also of the genes *FUT8, MGAT3* and *B4GALT* in the CG combined group (r_s_ = 0.418, 0.643, 0.534 respectively, *p* < 0.003) consistent with the shared common *N*-glycan synthesis pathway.

On account of the known population variation (SNPs) for *MGAT3*, *FUT8* and in the case of possible variation in expression of the *ALG9* gene we compared the fold differences in expression between these genes in the Irish and Dutch cohort. There was no statistically significant difference in expression noted.

More than 50% of all proteins are considered to be glycosylated in humans. Glycan oligosaccharide modification affects receptor function, cell signalling, and cell adhesion. Modification of branched *N*-glycans structures such as bisecting GlcNAc, β-1,6-GlcNAc and core fucose (α-1,6-fucose), the enzymatic products of *MGAT3* and *FUT6* genes, shown to be abnormal in galactosaemia [9], are highly associated with biological functions involving cell adhesion [34, 36].

Almost all key molecules involved in innate and adaptive immune responses are glycoproteins. IgG is the most abundant circulating glycoprotein (as measured in this study). The addition of different glycans to its Fc regions (region that interacts with cell surface receptors) and changes in core fucosylation can have dramatic effects on its effector function [37, 38].

In addition to *N*-glycan synthesis genes in this study, we thus sought to examine whether there was also an identifiable anti-inflammatory component to the pathophysiology of galactosaemia. We proposed from our earlier studies that systemic glycosylation abnormalities evidenced in galactosaemia could be associated with abnormal inflammatory responses. We studied two genes as possible anti-inflammatory markers, *ANXA1* and *ICAM1,* the former which we previously reported as dysregulated in galactosaemia [26]*.*

*ANXA1* (Annexin A1) is a phospholipid binding protein which responds to a glucocorticoids and carries out an anti-inflammatory response [39]. The expression of *ANXA1* was increased 6.2 fold in the CG combined group females vs healthy controls, (*p* < 0.01), which may reflect upregulated apoptosis pathways, one of the proposed possible mechanisms of primordial follicle depletion.

Another consideration is an anti-inflammatory response to the sub-galactosylation of immunoglobulins resulting in increased immune activation as shown for IgG, whereby reduced galactosylation of the Fc region results in an inflammatory conformation [40].

*ICAM1 (*Intercellular Adhesion Molecule 1*)* encodes a cell surface glycoprotein which is typically expressed on endothelial cells and cells of the immune system which binds to integrins. *ICAM1*’s concentration greatly increases upon cytokine stimulation [41]. *ICAM1* was observed to be upregulated 4.79 fold in the CG combined group (*p* < 0.01). The expression of *ANXA1* and *ICAM1* expression correlated strongly in galactosaemia patients (r_s_ = 0.624, *p* < 0.05 × 10^− 4^), suggesting an inflammatory association.

*ANXA1* correlated strongly with expression of the glycan assembly gene *ALG9* in the CG combined group (r_s_ = 0.840, *p* < 0.05 × 10^− 12^). Also ncreased expression of *ICAM1* correlated with the increased expression of *ALG9* in the CG combined group (r_s_ = 0.615, *p* < 0.05 × 10^− 4^) indicating a possible relationship between inflammation and abnormal glycosylation in these patients.

*SEPT4* (the gene that encodes Septin 4) was increased in expression 3.6 fold (*p* < 0.005). The septin family of proteins are a group of GTP-binding proteins that are essential for biological processes such as cytokinesis and vesical trafficking. *SEPT4* has been shown to be involved with sperm terminal differentiation in mice and is a marker of apoptosis, required for the induction of cell death mediated by TGF-beta and by other apoptotic stimuli [42].

In addition to the identification of dysregulation of specific genes in CG, we and others have proposed that abnormal glycosylation leads to systemic signalling abnormalities. We noted significant dysregulation of the leptin receptor (*LEPR)* in our earlier studies and also reported decreased circulating serum leptin in both CG males and females [27]*.* We now report dysregulated expression of leptin *(LEP)* and *LEPR* in both CG males and females in the present extended study and provide a link between glycosylation (G-ratio) and leptin expression. Both *LEP* and its receptor (*LEPR*) were upregulated 8.8 and 6.38 fold respectively (*p* < 0.005) in the CG combined group (male and females). The fact that we noted upregulation of the leptin gene in CG male and female PBMCs but reduced serum concentration in both CG males and females (not statistically significant in females) requires further investigation.

The finding of normal leptin circulating concentrations in females as previously identified may reflect the fact that the majority of females in the study who manifested POI were on hormone replacement therapy as reviewed earlier in our previous study [27].

### Biochemical and endocrine clinical correlates with gene expression

We noted that Gal-1-P levels correlate positively with G-ratios which may be consistent with a recent observation of better clinical outcomes in male and female CG patients who exhibit lower RBC Gal-1-P levels on a galactose restricted diet [17]. Lower Gal-1-P levels with lower G-ratios may be explained by residual GALT activity, accessory pathway metabolism and less endogenous turnover of galactose from sources such as glycoproteins [43].

Testosterone levels have been noted to be in the lower normal range in a small number of galactosaemic males but within reference ranges [33]. Testosterone levels in this patient cohort were within the normal reference range (Table 2). As expected with the normal values for FSH and LH observed in our male cohort, there was no correlation noted between *G*-ratios and FSH and LH levels in males (data not shown). LH, as an indicator of POI in females, was shown to correlate positively with both abnormal G0/G1 and G0/G2 ratios in females.

Leptin levels (BMI corrected) were shown to correlate negatively with G-ratios in the CG combined group. Leptin levels correlated positively with the hormone FSH in the combined cohort and with testosterone in males.

Leptin is an important hormone predominantly released by adipocytes which has key roles in the regulation of energy balance, body weight, metabolism, neuroendocrine function, reproductive function and bone formation. Leptin exhibits its activity by binding to its fully active receptor (Ob-R, encoded by *LEPR)* which activates the JAK2 signalling mechanism and activators of transcription (STATs) [44–46] (see Fig. 4).

Reproductive signals integrate at different levels of the hypothalamic-pituitary-gonadal (HPG) axis involving GnRH, the pituitary hormones LH and FSH and gonadal hormones (see Fig. 4). Hypoleptinemia associated with energy deficiency influences several neuroendocrine axes including the thyroid, gonadal, cortisol and growth hormone axes. The identification of humans with mutations of the leptin and leptin receptor gene has illustrated how leptin deficiency influences the onset of puberty [47]. Leptin replacement has been shown to result in resumption in ovulation, increase in LH and oestradiol levels in blood and increase in follicular diameter and number in women with hypothalamic amenorrhea and replacement of leptin in deficient individuals has led to the successful treatment of hypogonadism by gonadotropin secretion and the restoration of puberty and fertility [48].

Although the primary site of leptin is the control of the HPG axis in the brain the actions of leptin have been shown on other reproductive systems including the gonads. Leptin expression has been confirmed in ovarian granulosa and cumulus cells and in mature human oocytes [49]. The leptin receptor also has been shown to be expressed in theca and granulosa cells on the human ovary with a putative function of leptin in the ovary to control folliculogenesis [50]. Expression of leptin receptors has also been reported in the testis [51].

Also leptin has a pro-inflammatory effect stimulating T Lymphocyte proliferation and cytokine production and is proposed to be an important link between nutritional status and the immune system [52]. LEPR activity on astrocytes has been proposed to actively regulate leptin transport across the blood brain barrier, a finding consistent with evidence that central regulatory changes of LepR during obesity and inflammation often occur in astrocytes [53, 54].

In our studies in Galactosaemia, we have identified a primary alteration of the leptin system in CG patients with low circulating leptin levels in males and females which are statistically significant in males with the expected correlation between log leptin and BMI evident in CG females who were mostly on HRT but not in CG males. The corresponding gene expression, the *LEP* gene and its receptor is significantly overexpressed in galactosaemia vs healthy controls (upregulated 8.88 fold, *p* < 0.01 and 6.38 fold, *p* < 0.001, respectively). Both the leptin gene *LEP* and its receptor gene *LEPR* are upregulated in CG males (*n* = 27) and CG females (*n* = 27).

From our earlier studies, we have suggested that abnormal *N*-glycosylation could putatively disrupt leptin-HPG signalling resulting from distorted Ob-r, soluble leptin receptor (sOb-R) and GnRH receptor (GnRH-R) function in galactosaemia [53].

Leptin exerts paracrine effects and is predominantly synthesized in adipose tissue. Expression of *LEP* gene is weight-course dependent and circulating leptin concentrations can be indicative of an adaptive pattern of *LEP* gene expression in obese subjects undergoing weight reduction [55]. Conversely, expression of *LEPR* gene, which is abundantly present in adipose tissue specimens from lean subjects, is found reduced in specimens obtained from obese patients [56]. As we could not examine adipose tissue specimens as part of this study it is unclear if the upregulation of expression of *LEP* and *LEPR* observed in white blood cells from this Galactosaemia cohort with the existence of decreased circulating leptin levels relates to altered transcriptional regulatory pathways, inflammatory mediators, or adaptive changes directly affecting leptin signalling molecules such as Suppressor of Cytokine Signaling 3 (SOCS3) [56].

The role and context of *ANXA1* as an anti-inflammatory and apoptosis marker in females requires further elaboration. Primordial ovarian follicular atresia and dysgenesis can be noted in the first year of life in affected galactosaemia females. It is considered that abnormal signalling for development of this dysgenesis may occur prenatally and during the first years of life linked to an apoptosis pathway. It has recently been demonstrated that human ovarian explants exposed to ibuprofen (anti-inflammatory) showed reduced cell number, less proliferating cells, increased apoptosis and a dramatic loss of germ cell number [57]. Whether *ANXA1 i*s a marker of apoptosis in this pathway remains to be determined.

Limitations of the study: The findings of this study involve a limited number of adult CG patients. As with many rare diseases it can be challenging to recruit large sample sizes, in particular for adult patients when attendance to clinic may be lower than anticipated. Further validation of the significant gene expression abnormalities could be indicated.

In addition, these data relate to a Dutch and Irish population. The population frequency of modifying SNPs for the glycan synthesis genes *ALG9, MGAT3, FUT8,* and *B4GALT1* for the Dutch and Irish populations in comparison to other populations is currently not known. However, we did not note any statistical differences in gene expression differences between the Dutch and Irish study groups for these glycan synthesis genes. Also, as glycosylation is determined not only by genetic control but very significantly by epigenetic effects, variations in environmental influences including changes in dietary substrate (glucose and galactose exposure) would be expected to modify the specific glycan gene expression between subjects on an individual basis dependent on environmental exposures.

This study describes the gene expression in peripheral blood lymphocytes in adults with CG compared with N-glycosylation and endocrine hormonal measurements. The findings cannot describe the ovarian specific tissue expression or prenatal developmental expression or aberrant signalling effects presumed to be in existence prenatally with evidence of profound serum *N*-glycome intoxication effects (assembly defects) in CG intoxicated neonates with CG (25).

## Conclusion

In this study we have demonstrated the validation of specific key *N*-glycan synthesis genes, and related genes associated with inflammation and fertility in CG patients. These findings were correlated biochemically with IgG *N-*glycan galactose incorporating phenotypic markers and also with phenotyping endocrine markers of POI in females and fertility in males. As shown in Fig. 4, this study of galactosaemia has illustrated the dysregulation of glycosylation, inflammatory response and leptin metabolism as possible cellular event sequences with apoptosis in CG. These studies further illustrate the complex nature of the CG phenotype, in particular in relation to fertility. We propose also that these studies of a rare inborn error of metabolism involving central *N*-glycosylation have also illustrated how significant oligosaccharide modification/glycosylation ‘modifier’ epigenetic gene effects involving recently described glycan modifier genes influencing cell signaling converts Galactosaemia and its outcome analysis from a simple mendelian disease to a complex trait, applicable to other related disorders [10, 11].

## Methods

### Study subjects and characterisation

For this multi-center cross sectional study, RNA samples from 54 adult CG adult patients were included for gene expression analysis. This included 36 Irish patients and 18 Dutch patients (age range 17–51). The genotypes and gender of these subjects are illustrated in Table 1.

The inclusion criterion was a diagnosis of CG (confirmed by the genotypes illustrated in Table 1 or by the presence of absent or virtually absent GALT enzyme activity). All study patients were maintained on a dietary galactose intake of < 1000 mg galactose/day. All patients entered in the study were compliant with regular clinic attendances and adherence to the galactose dietary restriction. Recent biochemical monitoring by RBC Gal-1-P in this adult population was available for only a subset of subjects. 16 apparently healthy adult control subjects aged between 20 and 40 years (9 females and 7 males) provided RNA samples as healthy controls.

### Endocrine studies/hormonal assays

FSH, LH, and leptin were analysed in females and males. Oestradiol was measured in females and testosterone in male subjects. FSH, LH and oestradiol were measured by chemiluminescent immunoassay on the Abbott Architect i2000SR (Abbott Diagnostics, Illinois, USA). Between run coefficients of variation (CV) for LH and FSH were < 5%, while the maximum between run CV for oestradiol was 6.3% at 147 pmol/L. The assay limits of quantitation were 0.5 IU/L, 0.5 IU/L and 92 pmol/L for LH, FSH and oestradiol respectively. Testosterone was analysed using liquid chromatography tandem mass spectrometry (LC-MS/MS using a Waters Acquity UPLC coupled to a Xevo TQ tandem quadrupole using positive ion electrospray MS and multiple reaction monitoring (MRM). Serum leptin was measured using a human leptin immunoassay: an in-house immunoassay using DELFIA technology with antibodies and standards purchased from R&D Systems (R&D Systems Europe, Abington UK). CVs were 7.1% at 2.7 ng/mL, 3.9% at 14.9 ng/mL and 5.7% at 54.9 ng/mL (in-house data) and the Lower Limit of Detection (LLOQ) was 0.1 ng/mL. The concentration of sOb-R was determined using a commercially available ligand immunofunctional assay with reagents and standards purchased from R&D Systems. CVs ranged from 5.3 to 8.6% and the Lower Limit of Detection was 0.7 ng/mL.

### IgG *N*-glycan analysis

The method of IgG glycoprofiling was as previously described [22, 23]*.* Briefly, serum was extracted from whole blood and immediately frozen. IgG was captured and eluted with Protein G spin plates. IgG was then denatured and *N*-glycans were released with Peptide *N*-glycosidase F (PNGase F). *N*-glycans were washed and labelled with anthranilamide (2-AB) and analysed on a Waters Acquity UPLC machine. The ratios of non-galactosylated, mono-galactosylated and di-galactosylated *N*-glycan species were compared (G-ratios)]. Statistical analysis was performed as described below with SPSS software (IBM).

### TaqMan qPCR arrays

Within 3 h of collection, the whole blood samples were spun in BD Vacutainer cell preparation tubes (Fisher Scientific, Loughborough, UK), which allow rapid isolation of PBMCs. Total RNA was extracted from PBMCs using the RNeasy Plus Mini Kit (Qiagen Ltd., Manchester UK). cDNA was produced following reverse transcription of total RNA using the RT2 First Strand Kit (Qiagen Ltd).

Custom format ABI Taqman Array plates (Applied Biosystems, Foster City, CA, USA), were manufactured for 12 selected gene probes; *ALG9, ANXA1, B4GALT1, FUT8, ICAM1, LEP, LEPR, MGAT1, MGAT3, SEPT4, UGDH* and *UGP2* along with three selected controls; *ACTB*, *GAPDH* and *GUSB*, of which *ACTB* and *GUSB* were selected as reference genes for normalisation of target genes of interest. cDNA was applied to the customised plates and Quantitative Real-Time PCR analysis was performed on an ABI PRSIM 7900 HT Sequence Detection System with a 96-well Fast thermal cycling block (Applied Biosystems).

### Statistical analysis

For the RNA assays: Fold change of gene expression was quantified from raw CT scores with DataAssist software, version 3.01 using the 2^ΔΔCT^ method (Applied Biosystems). Genes with undetermined CT scores were excluded. Two control and three galactosaemia samples with undetermined CTs for the selected normaliser genes were excluded entirely. This was likely due to insufficient PBMC pellet or excessive RNA contamination in these samples. Next, the fold change values were tested for normality of distribution with Shapiro Wilk’s test and checked for homogeneity of variance with Levene’s test.

A *t*-test was not suitable as most of the genes were not normally distributed. We thus opted for a Mann Whitney U test which evaluates differences between the groups irrespective of normality or variance. Type 1 errors were controlled with application of the Benjamini–Hochberg False Discovery Rate using R-software, version 3.4.0.

Spearman’s rank correlation coefficient (r_s_) was used to evaluate correlating data. Preparation of boxplots and scatterplots, testing of statistical differences between groups and correlation tests were conducted with SPSS software, version 24 (IBM, New York, USA).

## Abbreviations

*ALG9*: Alpha-1,2-Mannosyltransferase
AMH: Anti Mullerian Hormone
*ANXA1*: Annexin A1
*B4GALT*: UDP-Gal:BetaGlcNAc Beta 1,4- Galactosyltransferase
cDNA: Complimentary Deoxyribonucleic acid
CV: Coefficient of Variance
ER: Endoplasmic reticulum
FSH: Follicle stimulating hormone
*FUT8*: Alpha-[1, 6]–fucosyltransferase
G0: Non-galactosylated
G1: Monogalactosylated
G2: Digalactosylated
Gal-1-P: Galactose-1-phosphate
GALE: Galactose epimerase
GALK: Galactokinase
GALT: Galactose-1-phosphate uridylyltransferase
GnRH: Gonadotropin Releasing Hormone
GP: Glycan peak
HILIC-UPLC: Hydrophilic Interaction Ultra-high Performance Liquid Chromatography
HRT: Hormone Replacement Therapy
*ICAM1*: Intercellular Adhesion Molecule 1
IMPase1: Inositol monophosphatase 1
*LEP*: Leptin
*LEPR*: Leptin receptor
LH: Luteinising hormone
*MGAT1*: Alpha-1,3-mannosyl-glycoprotein 2-beta-*N*-acetylglucosaminyltransferase
*MGAT3*: Beta-1,4-mannosyl-glycoprotein 4-beta-*N*-acetylglucosaminyltransferase
NS: Not significant
PNGase: Peptide *N*-glycosidase F
POI: Primary ovarian insufficiency
qRT-PCR: Quantitative real-time polymerase chain reaction
RBC: Red blood cell
RNA: Ribonucleic acid
SD: Standard deviation
*SEPT4*: Septin 4
sOb-R: Soluble leptin receptor
UDP: Uridine diphosphate
UGDH: UDP-Glucose 6-Dehydrogenase
UGP2: UDP-glucose pyrophosphorylase 2
UPR: Unfolded Protein Response
